# TXNDC12 and GDF2 as genetically supported plasma proteins associated with knee osteoarthritis: evidence from Mendelian randomization and preliminary biological support

**DOI:** 10.3389/fmed.2026.1865060

**Published:** 2026-07-08

**Authors:** Xiangyu Hu, Hao Rao, Li Luo, Shuang Tao, Gang Wu, Hanqiao Sun, Chao Li

**Affiliations:** 1 Hubei Provincial Institute of Traditional Chinese Medicine, Hubei Provincial Hospital of Traditional Chinese Medicine, Affiliated Hospital of Hubei University of Chinese Medicine, Wuhan, Hubei, China; 2 Hubei University of Chinese Medicine, Wuhan, Hubei, China

**Keywords:** FinnGen, Mendelian randomization, plasma proteins, preliminary biological support, primary knee osteoarthritis

## Abstract

**Background:**

Primary knee osteoarthritis (PKOA) is a common degenerative joint disease lacking effective disease-modifying therapies. Identifying circulating proteins associated with PKOA may provide clues for therapeutic target discovery.

**Methods:**

A two-sample Mendelian randomization (MR) study was conducted using protein quantitative trait locus data from deCODE Genetics, including 4,907 plasma proteins measured in 35,559 individuals, and PKOA genetic association data from the FinnGen study, comprising 18,563 cases and 363,345 controls. Single nucleotide polymorphisms were selected as instrumental variables, and sensitivity analyses were performed to assess the validity and robustness of the MR estimates. Bonferroni correction and the Benjamini- Hochberg procedure were applied for multiple testing correction. Protein–protein interaction, drug–gene interaction, and functional enrichment analyses were performed for prioritized proteins. Western blotting and enzyme-linked immunosorbent assay were used to provide preliminary biological support for MR-prioritized proteins.

**Results:**

Two plasma proteins showed genetic associations with PKOA susceptibility. Genetically predicted higher levels of thioredoxin domain-containing protein 12 (TXNDC12) were associated with an increased risk of PKOA (OR = 1.38, 95%CI: 1.27–1.50, *P* = 9.33 × 10^–15^), whereas growth/differentiation factor 2 (GDF2) was associated with a reduced risk (OR = 0.93, 95%: CI; 0.89–0.98, *P* = 4.17 × 10^–2^). After multiple testing correction, TXNDC12 remained significant after Bonferroni and false discovery rate (FDR) correction, while GDF2 met the FDR threshold only and should therefore be considered a hypothesis-generating finding requiring further validation. Network analyses identified 21 functionally related genes, including GSTA3, GSTM4, ACVRL1, and BMPR2. Preliminary experimental analyses showed increased TXNDC12 expression and decreased GDF2 expression in PKOA model mice compared with sham controls, consistent with the MR results.

**Conclusion:**

Our study identifies TXNDC12 as a genetically supported PKOA-associated plasma protein, while GDF2 remains a hypothesis-generating candidate requiring further validation. These findings provide preliminary associative evidence linking both proteins to PKOA and warrant further mechanistic investigation.

## 1 Introduction

Primary knee osteoarthritis (PKOA) is a common chronic degenerative joint disease characterized mainly by progressive cartilage degeneration and wear in the knee joint, accompanied by subchondral bone sclerosis or cystic changes and osteophyte formation at the joint margin (1, 2). Clinically, patients typically present with knee pain, stiffness, and restricted joint mobility, all of which can substantially compromise daily function and quality of life. PKOA represents a major global health burden, affecting approximately 16% of the population worldwide, with an estimated 86.7 million new cases reported in 2020. Age is one of its most important risk factors: the prevalence is approximately 10%–17% among individuals older than 40 years, rises to 50% in those older than 60 years, and may reach 80% in those older than 75 years (3). Although considerable progress has been made in understanding PKOA from pathological, molecular, and clinical perspectives, its underlying mechanisms remain incompletely defined. Current management relies largely on non-steroidal anti-inflammatory drugs (NSAIDs) and other symptomatic treatments, which may alleviate pain and improve joint symptoms to some extent but do not fundamentally prevent disease progression or recurrence. Prolonged dependence on these approaches often fails to achieve sustained disease control and may impose substantial physical, psychological, and economic burdens on patients, in addition to increasing healthcare costs (4). These challenges underscore the urgent need to identify novel targets and develop more effective intervention strategies for PKOA.

Plasma proteins are involved in a wide range of biological processes, including signal regulation, hormone homeostasis, molecular transport, coagulation and anticoagulation, immune responses, and nutritional support, and are essential for maintaining physiological stability (5). Because of their central biological roles, plasma proteins have attracted increasing attention as potential biomarkers and therapeutic targets across multiple diseases. In parallel, genome-wide association studies (GWAS) have greatly advanced the identification of disease-associated genetic variants and have provided important insights into disease biology. With the continued development of GWAS-based analytical frameworks, the integration of genomic and proteomic data has emerged as a powerful strategy for biomarker discovery and target prioritization. In this context, proteome-oriented genetic analyses offer a promising opportunity to investigate PKOA by helping to elucidate its complex molecular basis and to identify proteins that may be functionally involved in disease susceptibility and progression.

Mendelian randomization (MR) is a genetic epidemiological approach that uses genetic variants strongly associated with an exposure of interest, such as biomarkers or environmental factors, as instrumental variables (IVs) to infer potential causal relationships between exposures and disease outcomes (6). Owing to the random allocation of alleles during meiosis, MR is less vulnerable to reverse causation and can, to some extent, approximate the inferential framework of randomized controlled trials (RCTs). Because genetic variants are fixed at conception and are generally not influenced by subsequent environmental or behavioral factors, MR can also reduce confounding that commonly limits conventional observational studies (7). A valid MR analysis depends on three core assumptions: relevance, meaning that the instrumental variants are strongly associated with the exposure; (2) independence, meaning that they are not associated with confounders; and exclusion restriction, meaning that they influence the outcome only through the exposure. A recently published large-scale plasma proteome GWAS identified independent single nucleotide polymorphisms (SNPs) associated with thousands of plasma proteins, thereby providing an important genetic basis for protein quantitative trait locus (pQTL) studies (8). Building on this foundation, the present study integrated two-sample MR analysis, protein–protein interaction (PPI) network analysis, drug–gene interaction analysis, and functional enrichment visualization to investigate the functional relevance of potential pathogenic proteins in PKOA. Furthermore, based on the proteins prioritized by MR analysis, we performed WB and enzyme-linked immunosorbent assay (ELISA) to assess the expression levels of TXNDC12 and GDF2, providing preliminary biological support for these MR-prioritized proteins.

## 2 Materials and methods

### 2.1 Study design

**FIGURE 1:**
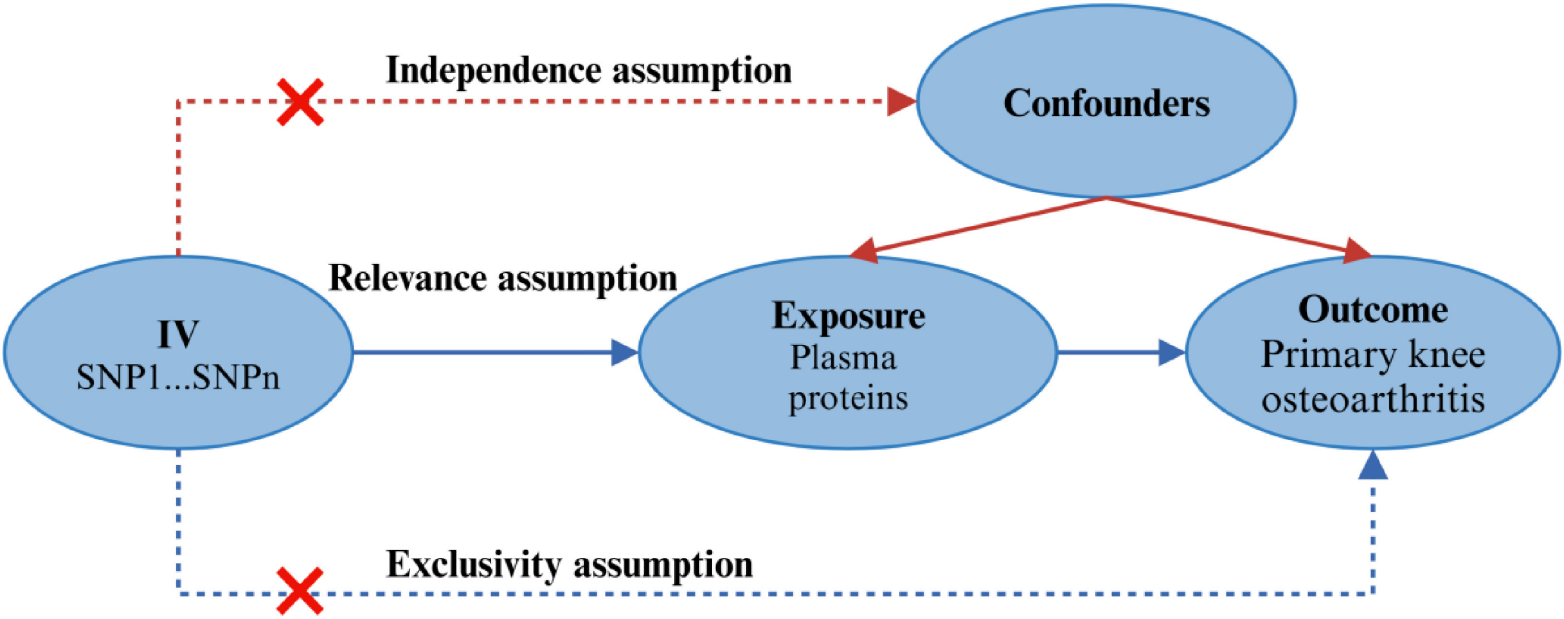
Overview and design of Mendelian randomization analysis. The analysis relies on three key assumptions: (1) Relevance assumption: SNPs are strongly associated with exposure (plasma proteins); (2) Independence assumption: SNPs are independent of confounding factors; (3) Exclusivity assumption: SNPs influence outcome (PKOA) exclusively through exposure (plasma proteins). IVs, Instrumental Variables; SNP, Single Nucleotide Polymorphism.

We conducted a two-sample MR analysis to investigate whether plasma proteins were causally associated with PKOA. Figure 1 summarizes the overall study workflow and illustrates the three core assumptions underlying MR analysis.

### 2.2 Data sources

The plasma protein data were obtained from deCODE Genetics, which measured 4,907 proteins in 35,559 individuals of European ancestry using the SomaScan platform^1^ (9). The GWAS summary statistics for plasma protein levels were derived from the deCODE Genetics study reported by Ferkingstad et al. (9). SomaScan is a high-throughput proteomic platform with high-sensitivity and precision, allowing the simultaneous quantification of a large numbers of proteins, including those with relatively low-abundance, and is therefore well suited for large-scale proteomic studies. The FinnGen study is a large-scale genomics initiative that has analyzed over 500,000 Finnish biobank samples and correlated genetic variation with health data to understand disease mechanisms and predispositions. The project is a collaboration between research organizations and biobanks within Finland and international industry partners. (The official website of FinnGen database requires the citation of this paragraph) Outcome data were derived from the FinnGen database^2^ (8). The FinnGen study includes up to 2,408 disease phenotypes and provides a valuable resource for biomarker discovery and molecular epidemiological research. In this study, GWAS summary statistics for PKOA were obtained from the FinnGen Data Release 10 (R10), using the endpoint R10_PRIM_KNEEARTHROSIS, which included 18,563 cases and 363,345 controls (Supplementary Table 1).

### 2.3 Mendelian randomization analysis

**FIGURE 2:**
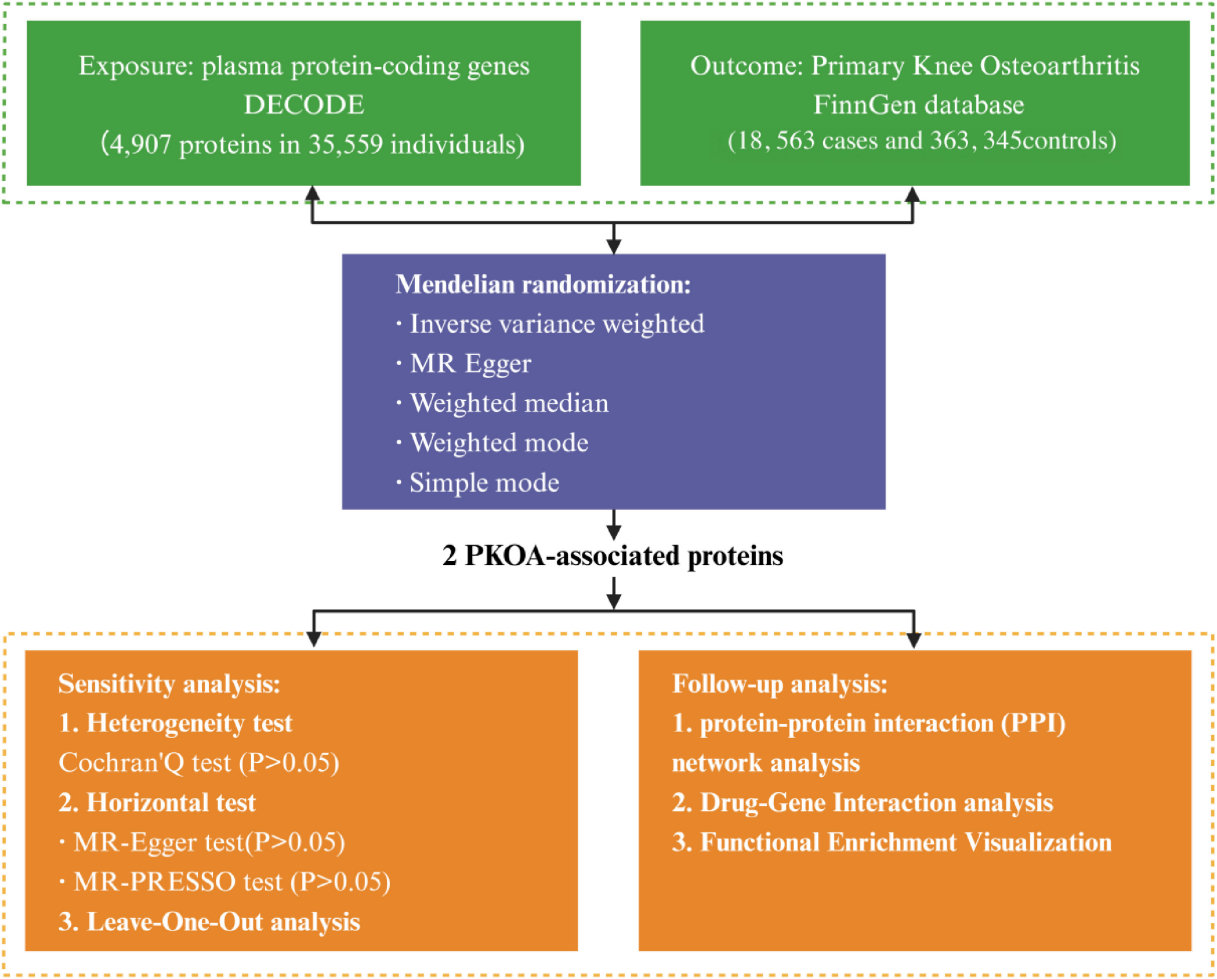
Flowchart of the study. PKOA, primary knee osteoarthritis. MR, Mendelian randomization.

Two-sample MR analysis was performed in R using the TwoSampleMR package to estimate the association between PKOA risk and genetically predicted protein levels (10, 11). Causal effects were evaluated using five complementary MR methods, including inverse variance weighted (IVW), MR-Egger, weighted median, simple mode, and weighted mode methods, with the IVW method regarded as the primary analytical approach (12). The IVW method assumes that all instrumental variables (IVs) are valid and satisfy the three core MR assumptions. By weighting each genetic variant according to the inverse of its variance, IVW provides an efficient pooled estimate of the causal effect. The MR-Egger regression was used to account for potential directional pleiotropy and can yield valid causal estimates even when some IV assumptions are violated (13). The weighted median method serves as a complementary approach and remains robust when at least 50% of the total weight is contributed by valid IVs (14). The simple mode method groups causal effect estimates and selects the most frequent cluster as the final estimate, thereby reducing the influence of outliers or invalid IVs. The weighted mode extends this approach by incorporating precision-based weighting, which can improve estimation stability. The odds ratio (OR) for PKOA was interpreted as the effect associated with a one-standard-deviation (SD) increase in plasma protein levels. Given that this study simultaneously conducts causal association tests for multiple plasma proteins, the Bonferroni correction was applied to control the family-wise error rate (FWER) in order to address the inflation of Type I errors resulting from multiple comparisons. Additionally, the Benjamini-Hochberg procedure was employed as a supplementary analysis to control the false discovery rate (FDR), with the FDR threshold set at 0.05. The overall MR framework is shown in Figure 2.

### 2.4 Sensitivity analysis

A series of sensitivity analyses was performed to assess the robustness of the MR findings. First, MR-Egger regression was used to evaluate horizontal pleiotropy (15), with the intercept term estimating its direction and magnitude; a *P*-value > 0.05 was considered to indicate no statistically significant evidence of horizontal pleiotropy. Second, heterogeneity among IVs was assessed using Cochran’s Q test, and *P* > 0.05 suggested no significant heterogeneity (16). Third, the MR-PRESSO method was applied to identify potential outlier IVs; a global test *P* > 0.05 suggested no significant evidence of horizontal pleiotropy-driven outliers. Finally, leave-one-out analysis was performed by sequentially removing each SNP and repeating the MR analysis to determine whether any single SNP disproportionately influenced the overall estimate (17).

### 2.5 Functional enrichment visualization

To explore the biological processes potentially linking plasma proteins to PKOA, we performed Gene Ontology (GO) enrichment analysis, Kyoto Encyclopedia of Genes and Genomes (KEGG) pathway analysis, and DISEASES enrichment analysis. The similarity threshold was set at 0.8. GO enrichment analysis was used to characterize associated biological processes, cellular components, and molecular functions within the identified gene sets (18). KEGG pathway enrichment analysis was conducted to clarify the involvement of these genes in defined biological pathways and their potential relevance to disease states. In addition, disease–gene association analysis integrates genomic data to identify genes and variants associated with specific diseases.

### 2.6 Protein-protein interaction network analysis and drug-gene interactions

We used the STRING database^3^ to construct a PPI network and to further investigate the relationships between potential targets and the pathophysiology of PKOA (19). The PPI network was generated using a minimum interaction score of 0.4 and visualized using Cytoscape software. In addition, we queried the DGIdb database^4^, which contains more than 14,000 drug–gene interactions involving approximately 2,600 genes and 6,300 drugs.

### 2.7 Ethics statement

All animal experiments was approved by the Laboratory Animal Welfare of Wuhan Lobin Life Science and Technology Company (LBSM2026047; approved on January 1, 2026). The study was conducted in accordance with local legislation and institutional requirements.

### 2.8 KOA-like model

Twelve mice underwent right knee surgery under sterile conditions. Pentobarbital sodium (50 mg/kg) was injected intraperitoneally. Anesthesia was induced by intraperitoneal injection of pentobarbital sodium at 50 mg/kg. Mice were randomly assigned to medial meniscus instability (DMM) group or sham operation control group. In the DMM surgery, the right knee joint capsule was opened, and the medial meniscotibial ligament (MMTL), which anchors the anterior horn of the medial meniscus to the tibial plateau, was carefully identified and transected, while the medial meniscus and all other supporting ligaments were kept intact. In the sham operation group, the joint capsule was opened to expose the MMTL, but the ligament was not transected. After surgery, the incision was closed layer by layer and disinfected daily with povidone iodine solution to prevent infection. Knee joint tissues and serum samples were collected 2 weeks after surgery for subsequent histological and molecular analyses. The successful induction of osteoarthritis in the DMM cohort was histologically verified before final inclusion in the study.

### 2.9 Histological confirmation of model establishment

Knee joints samples were harvested, fixed, decalcified, paraffin-embedded, and sectioned according to standard histological procedures. Safranin O/Fast Green staining was performed to evaluate articular cartilage integrity and proteoglycan content. Successful model establishment was defined by reduced or absent Safranin O staining, decreased proteoglycan content, surface irregularity of the articular cartilage, and disruption of cartilage architecture in the model group relative to the sham group. These histological features are consistent with the characteristic cartilage degeneration previously described in murine intra-articular fracture models.

### 2.10 WB analysis

To provide preliminary biological support for the MR-prioritized proteins, Western blotting was performed to assess TXNDC12 and GDF2 expression in sham and model groups. Independent biological replicates were included in each group (*n* = 3 per group). Total protein was extracted from each sample, separated by SDS-PAGE, and transferred onto polyvinylidene fluoride membranes. After blocking, the membranes were incubated overnight at 4 °C with primary antibodies against GDF2 (Thermo Fisher Scientific, A5-87966, rabbit polyclonal antibody, 1:1000), TXNDC12 (Thermo Fisher Scientific, MA5-55438, rabbit recombinant monoclonal antibody, clone 9J6L2, 1:1000), and β-actin (Thermo Fisher Scientific, AM4302, mouse monoclonal antibody, clone AC-15, 1:1000). The membranes were then incubated with HRP-conjugated goat anti-rabbit IgG (Thermo Fisher Scientific, 31460, 1:10000) or HRP-conjugated goat anti-mouse IgG (Thermo Fisher Scientific, 62-6520, 1:5000) at room temperature for 1 h. Protein bands were visualized using chemiluminescence. β-actin was used as the loading control, and relative protein expression was quantified by densitometric analysis and normalized to β-actin.

### 2.11 ELISA analysis

To further quantify protein-level changes, ELISA was performed to measure TXNDC12 and GDF2 levels in sham and model groups. Three independent biological replicates were included in each group, and each sample was measured in technical triplicate. GDF2 was measured using a Rat BMP-9 ELISA Kit PicoKine^®^ (Boster Biological Technology, EK1611; detection range: 15.6–1000 pg/mL; sensitivity: <10 pg/mL). TXNDC12 was measured using a Rat Thioredoxin Domain-Containing Protein 12 ELISA Kit (Abbexa, abx551902; detection range: 0.156–10 ng/mL). Standard curves and blank wells were included according to the manufacturers’ instructions. Optical density was recorded at 450 nm, and protein concentrations were calculated from the corresponding standard curves. The ELISA results provided additional quantitative assessment of the WB findings and were considered alongside the MR findings.

## 3 Results

### 3.1 Identification of plasma proteins associated with PKOA

**FIGURE 3:**
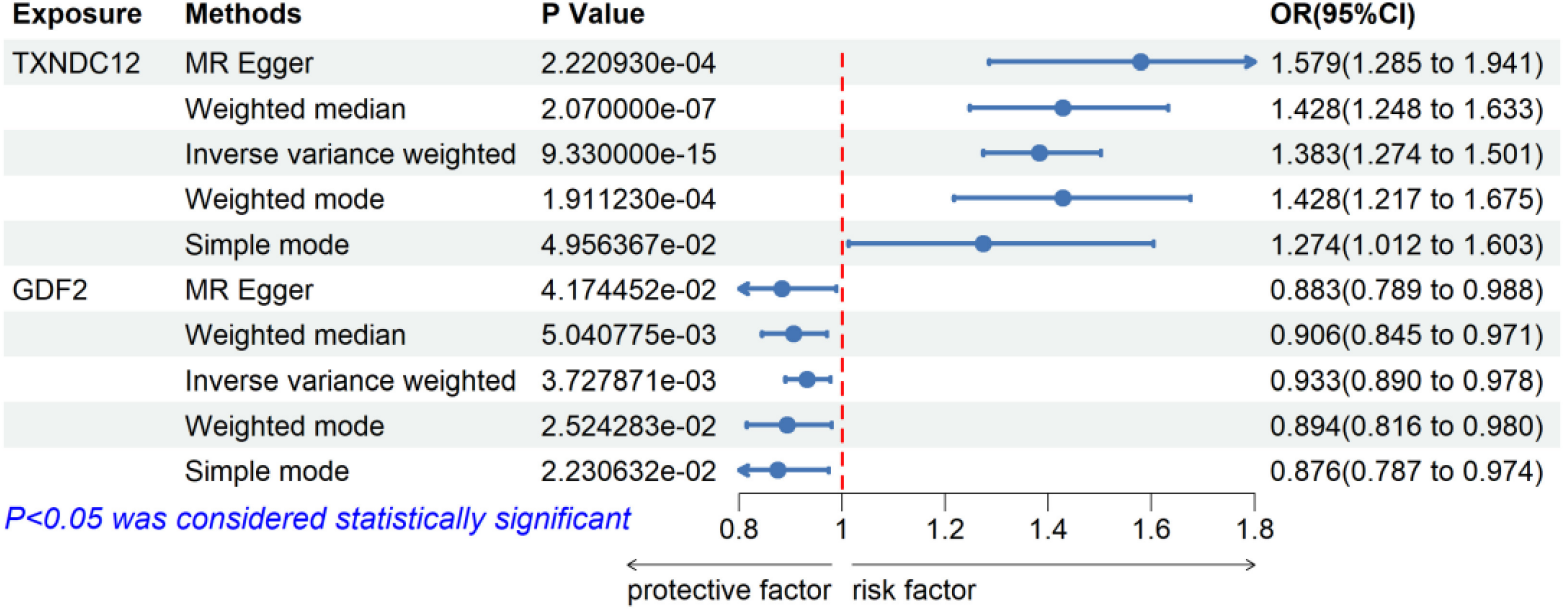
MR results for the genetic association between plasma proteins and PKOA is presented. MR, Mendelian randomization; OR, odds radio; 95% CI, 95% Confidence interval. Circles represent causal estimates, and lines represent the 95% CI for these ORs.

**FIGURE 4:**
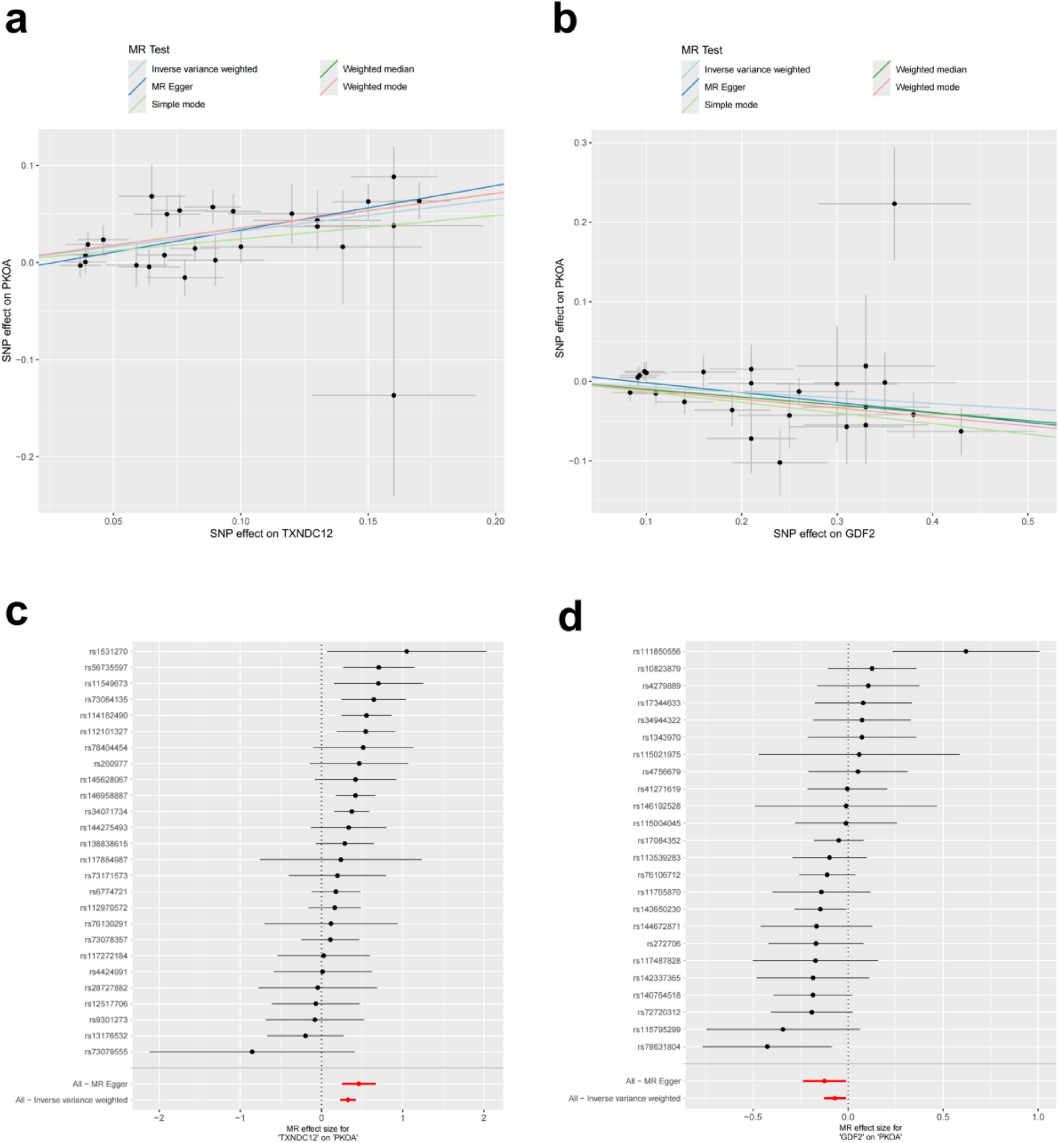
Scatter plots and forest plots of plasma proteins and the risk of PKOA. Scatter plots: Different regression lines represent effect sizes calculated through various MR tests. **(a)** Scatter plot for TXNDC12, **(b)** scatter plot for GDF2. Forest plots: Unidirectional causal inference for each SNP, with each black dot representing a single SNP. **(c)** Forest plot for TXNDC12, **(d)** forest plot for GDF2. MR: Mendelian randomization; PKOA: primary knee osteoarthritis; SNP: single nucleotide polymorphism.

Using five MR methods, including IVW, MR-Egger, weighted median, simple mode, and weighted mode, we initially identified 138 plasma proteins associated with PKOA when statistical significance was detected in any single method at *P* < 0.05 (Supplementary Figure 1). To improve robustness, we further prioritized proteins that showed consistent associations across multiple MR methods (*P* < 0.05) and excluded IVs exhibiting inconsistent causal directions or evidence of horizontal pleiotropy. After this multi-step filtering procedure, two plasma proteins were retained as key candidates: TXNDC12 and GDF2. Genetically predicted higher circulating levels of TXNDC12 were associated with an increased risk of PKOA across all MR methods (Inverse variance weighted: OR = 1.38, CI 1.27–1.50, *P* = 9.33 × 10^–15^; MR Egger: OR = 1.58, CI 1.28–1.94, *P* = 2.22 × 10^–4^; Weighted median: OR = 1.43, CI 1.25–1.63, *P* = 2.07 × 10^–7^; Weighted mode: OR = 1.43, CI 1.22–1.68, *P* = 1.91 × 10^–4^; Simple mode: OR = 1.27, CI 1.01–1.60, *P* = 4.96 × 10^–2^). In contrast, GDF2 showed a protective association, with higher genetically predicted levels linked to a reduced risk of PKOA (Inverse variance weighted: OR = 0.93, CI 0.89–0.98, *P* = 3.73 × 10^–3^; MR Egger: OR = 0.88, CI 0.79–0.99, *P* = 4.17 × 10^–2^; Weighted median: OR = 0.91, CI 0.85–0.97, *P* = 5.04 × 10^–3^; Weighted mode: OR = 0.89, CI 0.82–0.98, *P* = 2.52 × 10^–2^; Simple mode: OR = 0.88, CI 0.79–0.97, *P* = 2.23 × 10^–2^) (Figure 3 and Supplementary Tables 2–4). These associations were further visualized using scatter plots (Figures 4a, b) and forest plots (Figures 4c, d).

To further control the risk of false positives arising from multiple testing, this study applied Bonferroni correction and FDR correction to the two identified proteins. Following Bonferroni correction, the results using inverse variance weighted as the primary analytical method showed that TXNDC12 (Inverse variance weighted: Original *P* = 9.33 × 10^–15^, Bonferroni-adjusted *P* = 3.32 × 10^10^, FDR-adjusted *Q* = 4.67 × 10^14^; MR Egger: Original *P* = 2.22 × 10^–4^, Bonferroni-adjusted *P* > 0.05, FDR-adjusted *Q* = 3.33 × 10^–4^; Weighted median: Original *P* = 2.07 × 10^–7^, Bonferroni-adjusted *P* = 7.36 × 10^–3^, FDR-adjusted *Q* = 5.17 × 10^–7^; Weighted mode: Original *P* = 1.91 × 10^–4^, Bonferroni-adjusted *P* > 0.05, FDR-adjusted *Q* = 3.18 × 10^–4^; Simple mode: Original *P* = 4.96 × 10^–2^, Bonferroni-adjusted *P* > 0.05, FDR-adjusted *Q* = 4.96 × 10^–2^) was associated with an increased risk of PKOA. Both the Inverse variance weighted and Weighted median methods remained significant after Bonferroni correction (Bonferroni-adjusted *P* < 0.05). For TXNDC12, the FDR-adjusted *Q*-values were less than 0.001 under the four mainstream MR methods of Inverse variance weighted, MR Egger, Weighted median, and Weighted mode. The Simple mode method yielded an FDR-adjusted *Q*-value of 0.0496, which, although marginally below the conventional significance threshold of 0.05, is at a borderline significance level. This suggests that the result from this method has some reference value but requires cautious consideration. Nevertheless, all five MR methods showed consistent direction and magnitude of effect estimates, supporting the consistency of the genetically inferred association between TXNDC12 and PKOA (Supplementary Table 8). In contrast, GDF2 (Inverse variance weighted: Original *P* = 3.73 × 10^–3^, Bonferroni-adjusted *P* > 0.05, FDR-adjusted *Q* = 1.26 × 10^–2^; MR Egger: Original *P* = 4.17 × 10^–2^, Bonferroni-adjusted *P* > 0.05, FDR-adjusted *Q* = 4.63 × 10^–2^; Weighted median: Original *P* = 5.04 × 10^–3^, Bonferroni-adjusted *P* > 0.05, FDR-adjusted *Q* = 1.33 × 10^–2^; Weighted mode: Original *P* = 2.52 × 10^–2^, Bonferroni-adjusted *P* > 0.05, FDR-adjusted *Q* = 3.19 × 10^–2^; Simple mode: Original *P* = 2.23 × 10^–2^, Bonferroni-adjusted *P* > 0.05, FDR-adjusted *Q* = 3.16 × 10^–2^) was associated with a decreased risk of PKOA. Notably, none of the MR methods for GDF2 passed Bonferroni correction, yet all passed FDR correction (FDR-adjusted *Q* < 0.05), suggesting that this association may be influenced by multiple testing and should be interpreted with caution (Supplementary Table 8).

### 3.2 Sensitivity analysis of PKOA causative proteins

**FIGURE 5:**
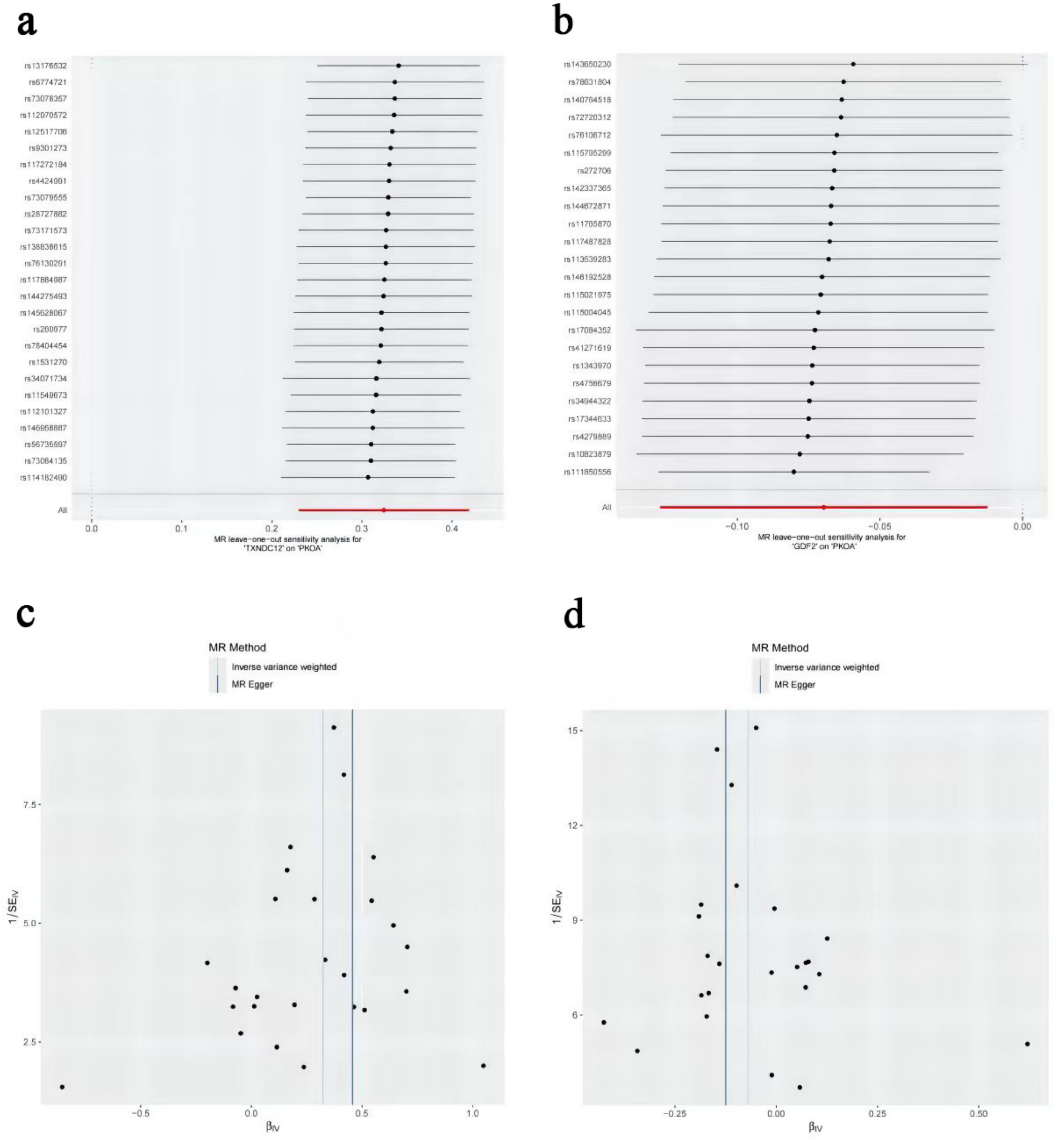
Sensitivity analysis results for identified PKOA pathogenic proteins. Leave-one-out sensitivity analysis: All SNPs represented by black dots are located on the same side of the “0” boundary line, indicating that no single SNP significantly interferes with the overall effect. **(a)** Leave-one-out sensitivity analysis for TXNDC12, **(b)** leave-one-out sensitivity analysis for GDF2. In the funnel plot, each point represents the effect size of a SNP. In the absence of bias, the funnel plot should display a symmetrical funnel shape, indicating no systematic relationship between the precision of the effect size and the effect size itself. **(c)** Funnel plot for TXNDC12, **(d)** funnel plot for GDF2. PKOA, primary knee osteoarthritis; MR, Mendelian randomization; SNP, single nucleotide polymorphism; β, effect estimate; SE, standard error.

A series of sensitivity analyses were conducted to evaluate the robustness of the MR findings (Supplementary Tables 1, 2). Cochran’s Q test under the IVW model indicated no significant heterogeneity among the instrumental variables for either TXNDC12 (26 SNPs) or GDF2 (24 SNPs). In addition, the MR-Egger intercept testing (*P* for intercept > 0.05) and the MR-PRESSO global test (*P* for global test > 0.05) did not detect no significant horizontal pleiotropy, suggesting that the causal estimates were unlikely to be influenced by directional pleiotropic effects. Leave-one-out analysis showed that no single SNP materially altered the overall causal estimates after sequential exclusion (Figures 5a, b). In addition, funnel plots visually suggested good symmetry in the SNP-specific effect estimates, with no obvious outliers or anomalies, further supporting the stability and reliability of the causal estimates (Figures 5c, d). Collectively, these results suggest that the identified associations between the prioritized proteins and PKOA were unlikely to be driven by major confounding or pleiotropic bias. Therefore, our findings suggest that the causal relationships between the identified proteins and PKOA are not confounded by potential risk factors. After multiple testing correction, the genetically inferred association between TXNDC12 and PKOA remained statistically significant across the primary MR methods. In contrast, while the association for GDF2 passed FDR correction, it did not withstand Bonferroni correction and should therefore be interpreted with caution.

### 3.3 Functional enrichment visualization analysis

**FIGURE 6:**
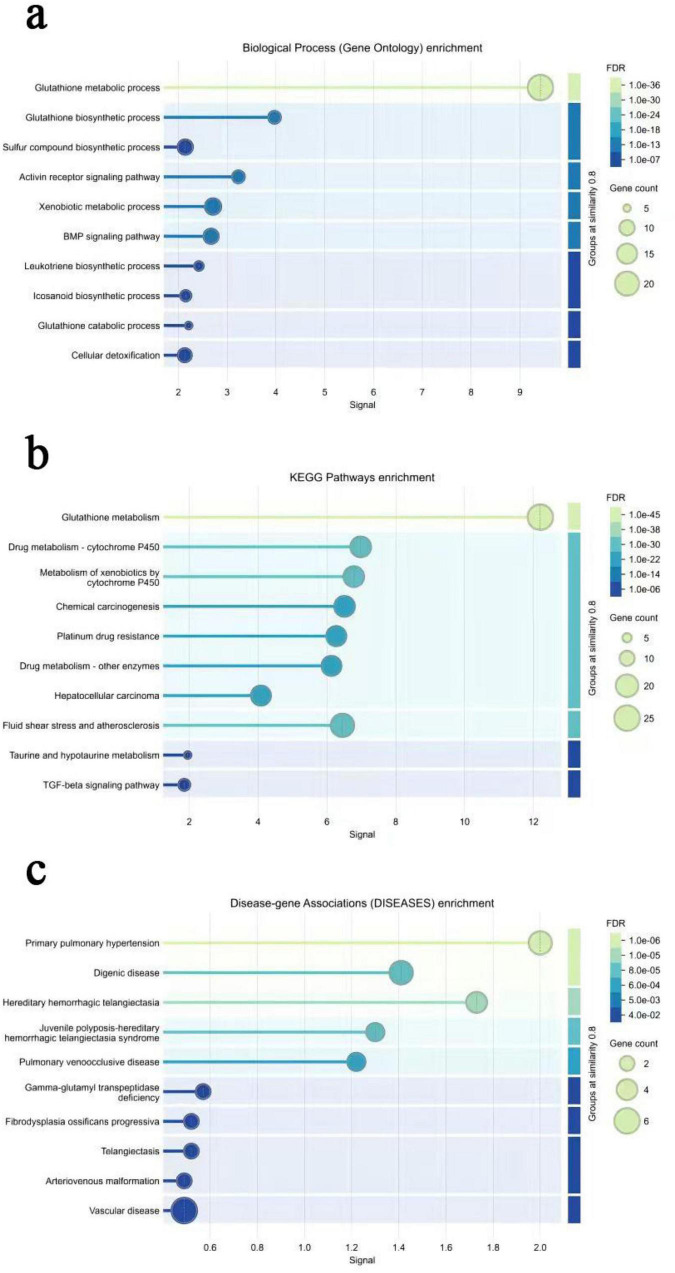
Functional enrichment visualization. KEGG, Kyoto Encyclopedia of Genes and Genomes. FDR, false discovery rate. The color intensity indicates the strength of the enrichment signal, with lighter colors representing lower FDR values, stronger enrichment, and higher statistical significance; the size of the circles represents the number of genes involved in the biological process, with larger circles indicating a greater number of genes participating in the process. **(a)** Biological process (Gene Ontology) enrichment, **(b)** KEGG pathways enrichment, **(c)** disease-gene associations (DISEASES) enrichment.

Through integrated multi-layer bioinformatics analyses, we found that TXNDC12 and GDF2 were potentially related to several biological processes and disease-related pathways. GO and KEGG enrichment analyses showed that both proteins were significantly enriched in pathways related to glutathione metabolism, sulfur compound biosynthesis, and drug metabolism-cytochrome P450. These processes are important for maintaining cellular redox homeostasis, xenobiotic metabolism, and protection against chemical stress. Notably, glutathione metabolic processes were consistently enriched in both GO and KEGG analyses, highlighting a possible association between these proteins and antioxidant defense mechanisms. Disease-gene association analysis using the DISEASES database further revealed potential links between TXNDC12/GDF2 and several vascular diseases, including primary pulmonary arterial hypertension, hereditary hemorrhagic telangiectasia, and pulmonary veno-occlusive disease. Together with previously reported links to fibrodysplasia ossificans progressiva and telangiectatic phenotypes, these findings suggest that TXNDC12 and GDF2 may be involved in or reflect biological processes related to vascular remodeling, blood pressure regulation, angiogenesis, and maintenance of vascular stability (Figure 6). Overall, these results suggest that TXNDC12 and GDF2 may represent putative molecular nodes associated with redox balance, detoxification-related metabolism, and tissue homeostasis, rather than providing definitive evidence of direct pathogenic roles.

### 3.4 PPI network analysis and drug-gene interaction results

**FIGURE 7:**
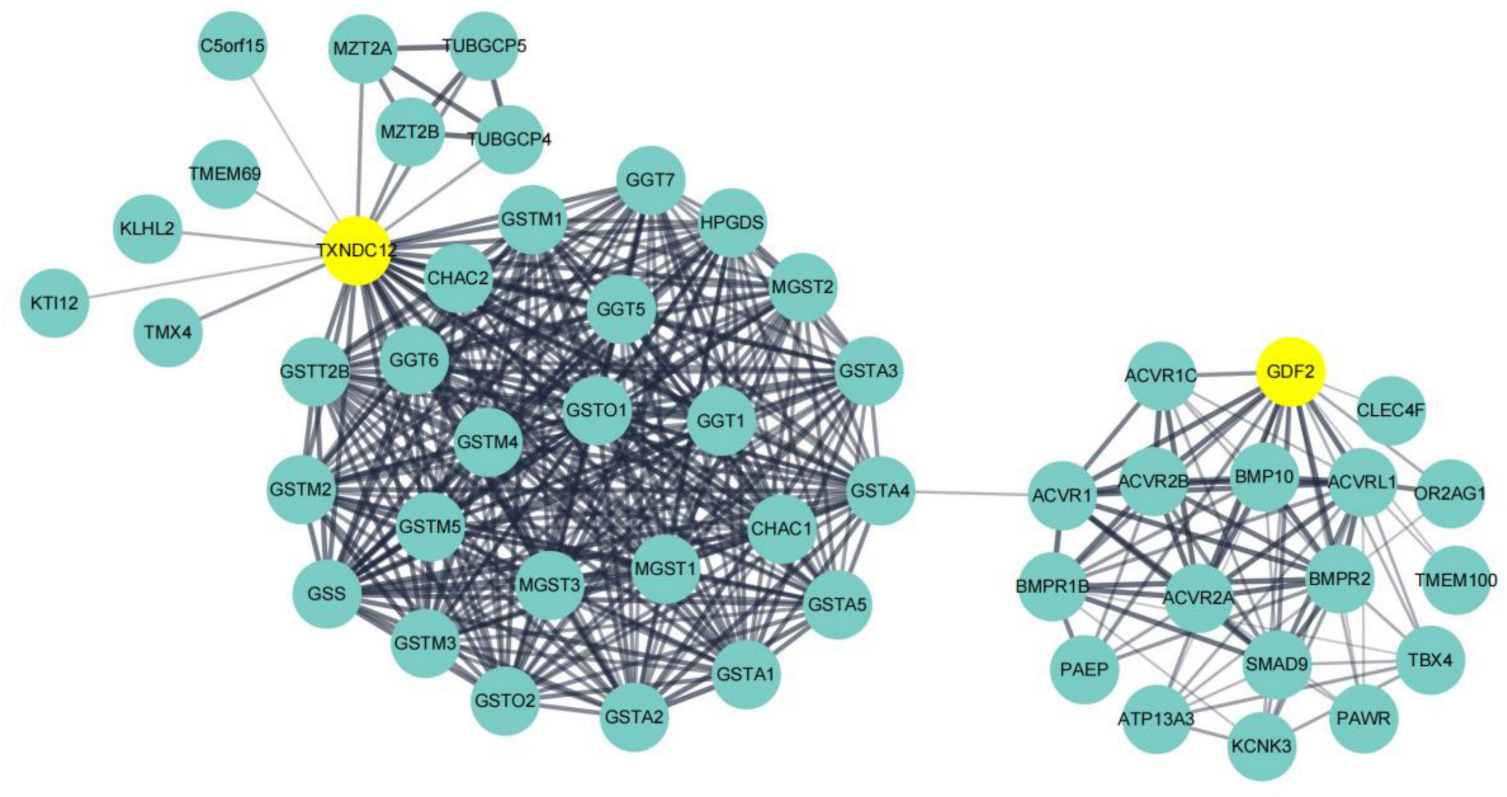
Protein-protein interaction network between identified protein targets and suggested protein targets. Yellow circles represent plasma proteins (TXNDC12 and GDF2), and green circles represent potential protein targets related to the currently identified proteins. The thickness of the black lines indicates the strength of the supporting data, with thicker lines representing stronger protein-protein associations.

To elucidate the functional context of our prioritized candidates, we constructed protein-protein interaction (PPI) networks for TXNDC12 and GDF2 using the STRING database. Through the CytoHubba ranking algorithm, we identified 33 proteins interacting with TXNDC12, including GSTA3, MGST3, GSTM4, GGT6, GGT7, GSTA4, GGT5, MGST1, GSTA5, GSS, GSTA2, GSTA1, GSTT2B, GSTO1, HPGDS, MGST2, CHAC2, GGT1, CHAC1, GSTO2, GSTM5, GSTM2, GSTM3, GSTM1, TUBGCP5, MZT2B, TMX4, MZT2A, TUBGCP4, KLHL2, TMEM69, KTI12, and C5orf15. In parallel, we identified 17 proteins interacting with GDF2, including ACVRL1, BMPR2, ACVR2A, ACVR2B, ACVR1, BMP10, SMAD9, BMPR1B, PAEP, ACVR1C, ATP13A3, OR2AG1, KCNK3, PAWR, TBX4, CLEC4F, and TMEM100 (Figure 7, Supplementary Figure 1). Integrating these topological insights with drug-gene interaction database, we mapped 21 potential targets within the combined 52-protein network. Specifically, 11 targets (GSTA3, GSTM4, GGT7, GSS, GSTA2, GSTA1, GSTO1, HPGDS, GGT1, GSTM3, and GSTM1) were linked to the TXNDC12-centered network, whereas 10 targets (ACVRL1, BMPR2, ACVR2A, ACVR2B, ACVR1, BMP10, BMPR1B, PAEP, ACVR1C, and KCNK3) were linked to the GDF2-centered network. These results provide a preliminary framework for future development of targeted strategies for PKOA (Supplementary Figures 2, 3 and Supplementary Table 6).

### 3.5 Histological validation of the PKOA model

**FIGURE 8:**
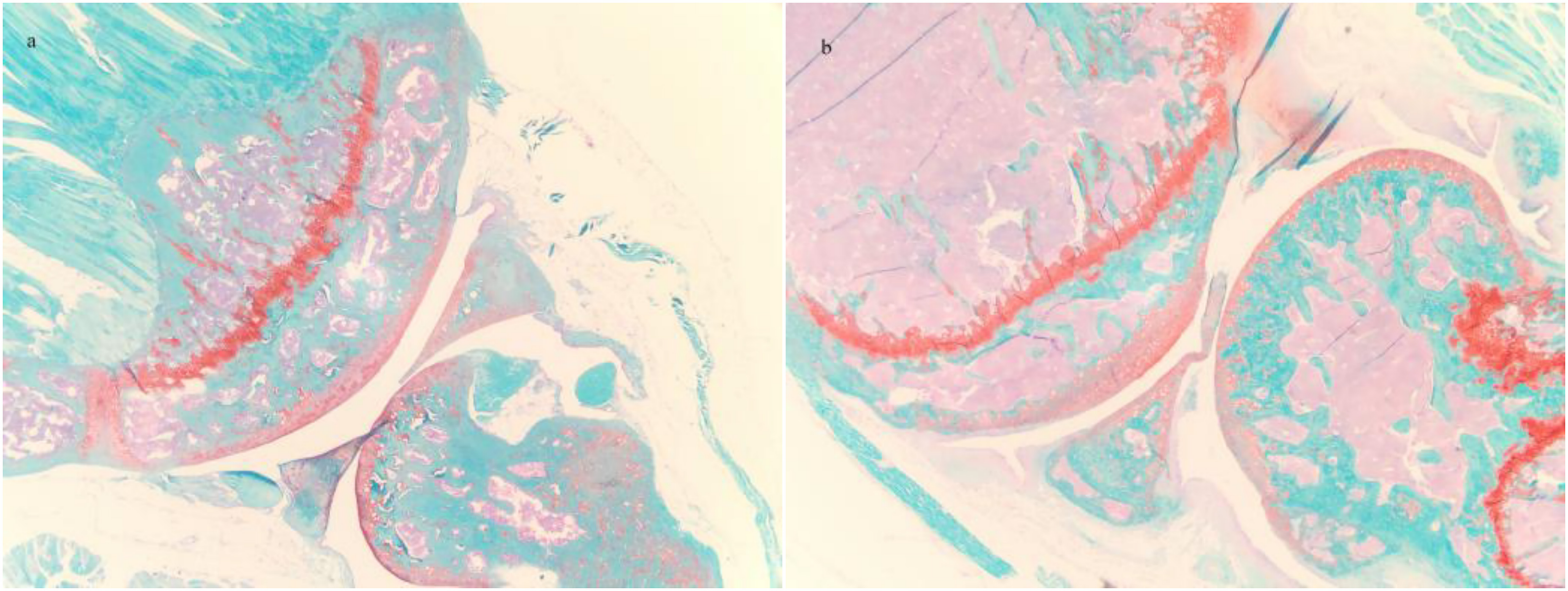
Safranin O–Fast Green staining. Safranin O - Fast Green staining of knee joints in sham and DMM groups. The sham group **(a)** showed a smooth and continuous articular cartilage surface with preserved Safranin O staining, whereas the DMM group, **(b)** exhibited reduced Safranin O staining, focal proteoglycan loss, and irregular cartilage surface, indicating OA-like histopathological changes.

Safranin O/Fast Green staining showed that articular cartilage in the sham group remained structurally intact with a smooth surface and preserved Safranin O staining, whereas the DMM) group exhibited reduced Safranin O staining, focal proteoglycan loss, surface irregularity, and cartilage structural disorganization, indicating successful induction of OA-like pathological changes in the mouse knee (Figure 8).

### 3.6 Preliminary biological support for TXNDC12 and GDF2

**FIGURE 9:**
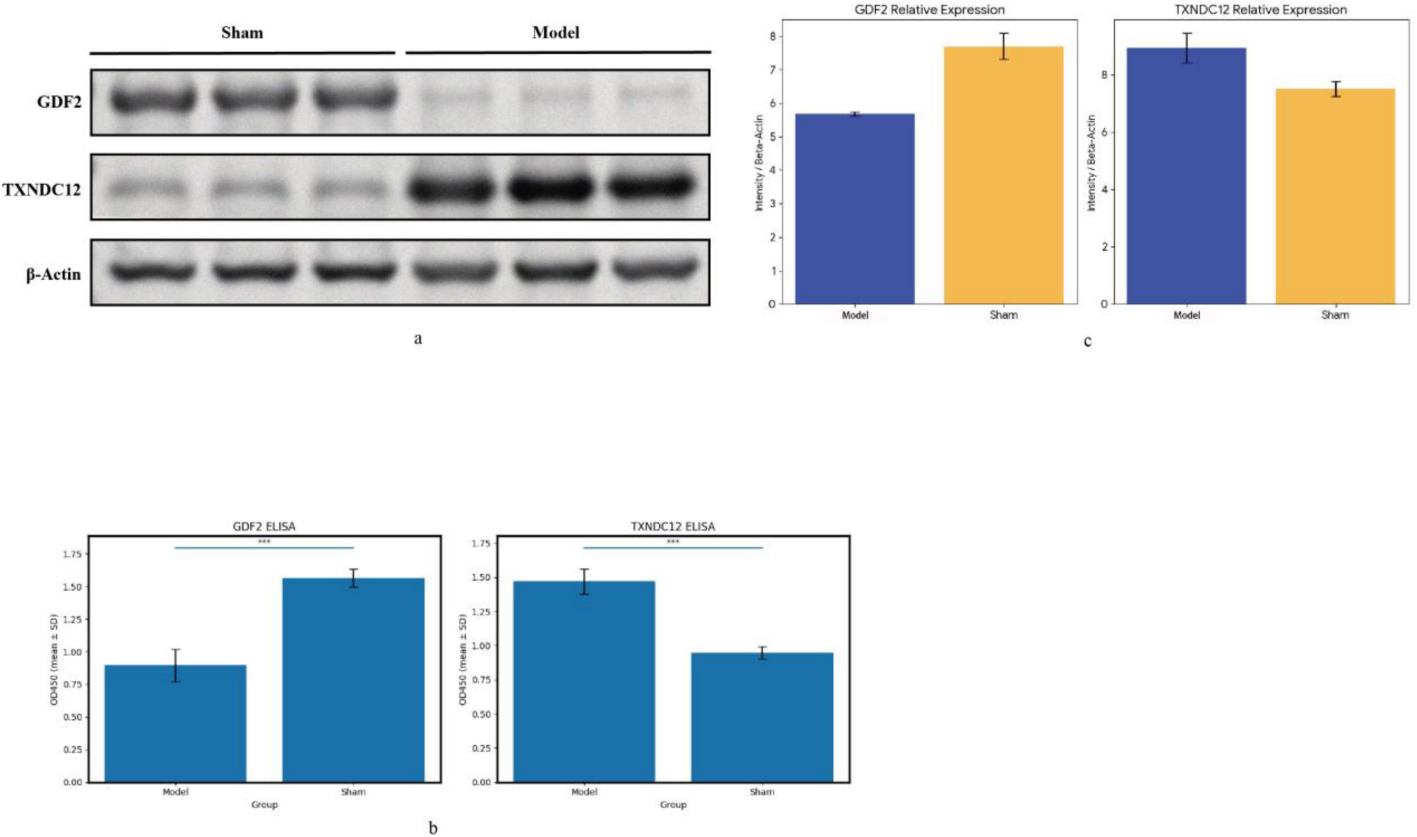
Western blot analysis and ELISA quantification. Western blot analysis of GDF2 and TXNDC12 protein expression in Sham and Normal groups **(a)**. Representative immunoblots show protein levels of GDF2 and TXNDC12, with β - Actin used as the loading control quantitative densitometric analysis normalized to β - Actin is presented on the right **(c)**. Data are expressed as mean ± SD from independent biological replicates. ELISA quantification of GDF2 and TXNDC12 protein levels in Medel and sham groups **(b)**. Bar graphs show mean OD450 values ± SD obtained biological replicates. Statistical significance between groups was determined using an unpaired two-tailed *t*-test; ***P < 0.001.

To provide preliminary biological support for the MR-prioritized proteins, we performed preliminary WB and ELISA analyses in sham and model groups. WB analysis showed that TXNDC12 protein expression was increased in the model group compared with the sham group, whereas GDF2 protein expression was decreased in the model group; β-actin levels remained stable across all samples, confirming equal loading. These trends were further corroborated by ELISA quantification, which detected elevated TXNDC12 and diminished GDF2 concentrations in the serum/tissue of model animals. Importantly, the results provided preliminary biological support for TXNDC12 as a genetically supported PKOA-associated protein and for a potential role of GDF2 in PKOA. Further validation is required to clarify the functional relevance of both proteins (Figure 9).

## 4 Discussion

In this study, we applied an integrative strategy combining two-sample MR, sensitivity analyses, bioinformatics annotation, and preliminary biological support through WB and ELISA analyses to explore the relationship between circulating plasma proteins and PKOA risk. Using plasma pQTL data from deCODE Genetics and PKOA GWAS summary statistics from FinnGen, we identified two proteins that were significantly associated with disease susceptibility. Genetically predicted higher levels of TXNDC12 were associated with an increased risk of PKOA, whereas higher GDF2 levels were associated with reduced risk. These associations were consistent across multiple MR methods and were supported by sensitivity analyses, indicating that the findings were unlikely to be driven by major pleiotropic bias. Similar MR-based approaches have also been applied in other disease contexts to infer genetically supported associations and prioritize biologically relevant targets, further supporting the utility of MR in exploring disease-related molecular mechanisms (3). Bioinformatics analyses further suggested that both proteins are linked to biological pathways involved in redox regulation and tissue homeostasis. Consistent with the genetic evidence, preliminary WB and ELISA analyses showed increased TXNDC12 and decreased GDF2 expression in the PKOA model group compared with the sham group. Together, these results prioritize TXNDC12 and GDF2 as putative PKOA-related proteins and provide preliminary insights into biological processes potentially associated with disease susceptibility. However, these experimental observations should be interpreted as preliminary and supportive rather than definitive evidence of causality.

Thioredoxin domain-containing protein 12 belongs to the thioredoxin (Trx)-like protein family and contains a conserved Trx domain involved in maintaining cellular redox balance (20, 21). Proteins in this family regulate oxidative protein folding and catalyze disulfide bond reduction, thereby contributing to intracellular antioxidant defense (22–24). And, previous studies have shown that oxidative stress-related signaling pathways, including NRF2-associated regulatory networks, are closely involved in cellular injury and inflammatory responses (25). Oxidative stress has long been recognized as a central mechanism in osteoarthritis progression (20, 26–28). Chondrocytes, the only cell type present in articular cartilage, are particularly sensitive to redox imbalance (29, 30). Excessive accumulation of reactive oxygen species can disrupt cellular homeostasis, promote extracellular matrix degradation, and induce chondrocyte senescence or apoptosis, ultimately accelerating cartilage degeneration (31–33). Stress-related cell death and organelle crosstalk may also contribute to pathological tissue remodeling and inflammation (34). In this context, the positive association between genetically predicted TXNDC12 levels and PKOA risk suggests that alterations in thioredoxin-related redox pathways may be associated with PKOA susceptibility (35–37). Our preliminary experimental findings showing increased TXNDC12 expression in the PKOA model further support its potential relevance to disease-associated oxidative stress responses. Nevertheless, given that thioredoxin family proteins are generally recognized for their antioxidant and cytoprotective functions, increased TXNDC12 expression should not be interpreted simply as evidence of a pathogenic role. Instead, this elevation may reflect a compensatory antioxidant response, a disease-associated molecular alteration, or a context-dependent biological effect in the joint microenvironment. Although the precise biological role of TXNDC12 in joint tissues remains unclear, these observations raise the possibility that TXNDC12 may be involved in PKOA-related redox imbalance or cartilage matrix remodeling, but further mechanistic studies are required to determine whether it has a direct functional role in disease progression.

Growth/differentiation factor 2, also known as bone morphogenetic protein 9 (BMP9), is a member of the TGF-β superfamily that plays important roles in vascular biology, skeletal development, and tissue homeostasis (38, 39). GDF2 exerts its biological effects through BMP receptor complexes, particularly by binding to the high-affinity receptor ALK1and activating Smad-dependent signaling pathways (38, 40). In addition to canonical Smad signaling, GDF2 can interact with multiple regulatory pathways, including Wnt, Notch, Hedgehog, and epidermal growth factor signaling, thereby influencing angiogenesis, tissue remodeling, and cellular differentiation (41, 42). In our study, genetically predicted higher GDF2 levels were associated with a reduced risk of PKOA, suggesting a putative protective association rather than definitive evidence of direct protection. This observation is consistent with the established functions of BMP family members in maintaining skeletal and connective tissue integrity. Moreover, preliminary WB and ELISA demonstrated lower GDF2 expression in the PKOA model group, further supporting the biological plausibility of its association with PKOA-related phenotypes. Although direct evidence linking GDF2 to PKOA remains limited, these findings suggest that GDF2 may be involved in maintaining joint tissue homeostasis or modulating inflammatory processes within the joint microenvironment. However, because the association between GDF2 and PKOA did not survive Bonferroni correction, the current evidence should be considered hypothesis-generating rather than definitive, and independent validation in additional cohorts is warranted.

Furthermore, multiple testing correction is critical to ensuring the reliability of discoveries. The simultaneous application of two correction strategies, including the Bonferroni correction for strict control of the family-wise error rate as the most conservative approach, and the FDR correction via the Benjamini-Hochberg procedure to control the proportion of false positive findings as a relatively lenient method, achieves a balance between statistical power and false positive control. Following stringent Bonferroni correction, only the association between TXNDC12 and PKOA remained significant, providing stronger statistical support for a genetically inferred association between TXNDC12 and PKOA. In contrast, although the association between GDF2 and PKOA demonstrated nominal statistical significance (IVW: *P* = 3.73 × 10^–3^), it did not reach the predefined significance threshold after Bonferroni correction. This result indicates that the causal relationship between GDF2 and PKOA requires validation in future cohorts with larger sample sizes. Nevertheless, GDF2 exhibited a consistently protective effect across all five MR methods (OR range 0.883–0.933), and sensitivity analyses revealed no evidence of horizontal pleiotropy or heterogeneity, findings that support the biological plausibility of GDF2 as a potential protective factor for PKOA. FDR correction results further demonstrated that all five methods for GDF2 achieved significance (FDR-adjusted *Q* < 0.05), indicating that this association retains a certain degree of statistical support under false discovery rate control. The discrepancy between the two correction methods reflects their distinct statistical properties. Bonferroni correction is excessively conservative, such that only extremely robust associations can surpass the threshold in large-scale testing, whereas FDR correction permits a certain proportion of false positives and offers greater statistical power in exploratory research. Although the raw *P*-value for GDF2 (IVW: *P* = 3.73 × 10^–3^) was substantially lower than the conventional significance level of 0.05, it remained approximately two orders of magnitude from the Bonferroni threshold, suggesting that its effect size or sample size may be insufficient to achieve significance under such stringent correction. This finding is consistent with recent reports from large-scale proteomic MR studies, which indicate that truly robust protein-disease associations are relatively rare following strict multiple testing correction (43–45). Importantly, previous MR studies have identified several plasma proteins associated with osteoarthritis; however, TXNDC12 and GDF2 have not been highlighted as major PKOA-associated proteins in these analyses. In addition, our study combined MR findings with WB and ELISA expression analyses, providing preliminary biological support for the MR-prioritized proteins. Therefore, TXNDC12 should be regarded as the protein with the strongest statistical support for association with PKOA susceptibility in the present study, given that it remained significant after both Bonferroni and FDR correction. In contrast, GDF2 should be considered a hypothesis-generating candidate, as its association was supported by FDR correction but did not survive the more stringent Bonferroni threshold. Although the consistency across MR methods and sensitivity analyses supports its biological plausibility, independent validation in additional cohorts and functional studies is required before firm conclusions can be drawn regarding its role in PKOA.

Several limitations should be acknowledged. First, the genetic datasets used in this study were derived predominantly from individuals of European ancestry, which may limit the generalizability of the findings to other populations. Second, although multiple sensitivity analyses were performed, MR relies on assumptions such as the absence of horizontal pleiotropy, which cannot be completely excluded. Third, differences in genotyping platforms, imputation strategies, and phenotype definitions across datasets may introduce additional heterogeneity. Fourth, the proteins investigated in this study were measured in plasma, which may not fully reflect the pathological status within the joint microenvironment. We did not have access to cartilage, synovial tissue, or synovial fluid proteomic data, and therefore could not determine whether the observed circulating protein alterations directly correspond to local intra-articular changes in PKOA. Finally, the WB and ELISA analyses in this study were preliminary and primarily demonstrated protein-level changes, which do not establish tissue-specific functional involvement or fully elucidate the downstream mechanisms of TXNDC12 and GDF2 in PKOA. These experimental analyses were limited to protein expression assessments using WB and ELISA and did not include mechanistic investigations. Moreover, the WB and ELISA analyses were performed with only three samples per group (*n* = 3), and therefore these findings should be interpreted as preliminary biological evidence. Overall, the current findings provide genetic and preliminary biological support only, and the functional involvement and therapeutic relevance of TXNDC12 and GDF2 in PKOA remain to be established. Although MR provides genetic evidence supporting genetically inferred causal associations, the WB and ELISA findings in the DMM mouse model remain correlational and descriptive. We did not perform loss-of-function or gain-of-function experiments, such as gene silencing, overexpression, specific inhibitor treatment, or pharmacological modulation, to determine whether targeting TXNDC12 or GDF2 can directly alleviate cartilage degradation or inflammatory responses. Therefore, TXNDC12 should be considered a prioritized candidate for further investigation, whereas GDF2 should currently be regarded as a hypothesis-generating candidate requiring additional validation. Further studies using cellular models, conditional knockout mice, intra-articular siRNA delivery, and well-characterized clinical cohorts are needed to clarify their functional roles and potential in PKOA. Furthermore, the FDR-adjusted *Q*-value for TXNDC12 under the Simple mode is 0.0496, indicating a borderline significant state. Although this value satisfies the conventional significance threshold (*Q* < 0.05), its proximity to the critical value suggests that the robustness of this association is relatively weak. Future validation in independent cohorts is warranted.

## 5 Conclusion

In summary, this study used a MR framework to systematically evaluate genetically supported associations between circulating plasma proteins and PKOA risk. TXNDC12 showed a genetically supported association with PKOA susceptibility, while GDF2 emerged as a hypothesis-generating candidate associated with disease risk. Preliminary experimental validation further demonstrated protein-level changes consistent with the MR findings, supporting the biological plausibility of these associations. These findings identify TXNDC12 as a putative PKOA-related protein and candidate biomarker, while providing preliminary associative evidence linking GDF2 to PKOA susceptibility. Further studies integrating genetic, molecular, and clinical evidence are required to confirm these findings and explore their translational relevance in PKOA prevention and treatment.

## Data Availability

The datasets presented in this study can be found in online repositories. The names of the repository/repositories and accession number(s) can be found in the article/Supplementary material.

## Footnotes

1
https://www.decode.com/summarydata/

2
https://r10.finngen.fi/

3
https://string-db.org

4
https://www.dgidb.org/

## Supplementary material

Supplementary Figure 1 Identification of 138 Plasma Proteins Significantly Associated with Primary Knee Osteoarthritis by Circular Heatmap Analysis.

Supplementary Figure 2 Protein-Protein Interaction (PPI) Network Connecting MR-Prioritized Proteins and Associated Protein Targets.

Supplementary Figure 3 Findings on the Types of Interactions in Drug - Gene Interaction Analysis.

Supplementary Table 1 Sources of Data for Mendelian Randomization Analysis in the Study.

Supplementary Table 2 Details for the SNPs related to plasma proteins.

Supplementary Table 3 Genetic instruments of PKOA for MR.

Supplementary Table 4 MR results for plasma proteins significantly associated with PKOA.

Supplementary Table 5 Results of Sensitivity Analysis for PKOA Pathogenic Proteins.

Supplementary Table 6 Results of Drug-Gene Interaction Analysis.

Supplementary Table 7 ELISA raw data and sample arrangement for GDF2 and TXNDC detection.

Supplementary Table 8 Mendelian Randomization Analysis and Multiple Testing Correction (Bonferroni Correction and FDR Correction) of Causal Associations Between Plasma Proteins TXNDC12 and GDF2 and PKOA.
