# Supplementary material for: TXNDC12 and GDF2 as genetically supported plasma proteins associated with knee osteoarthritis: evidence from Mendelian randomization and preliminary biological support
Source: Front Med (Lausanne). 2026 Jul 8;13:1865060. doi: 10.3389/fmed.2026.1865060 (PMC13389984; doi:10.3389/fmed.2026.1865060)
Supplement: Supplementary Figure 1 — Identification of 138 Plasma Proteins Significantly Associated with Primary Knee Osteoarthritis by Circular Heatmap Analysis. [file Data_Sheet_1.docx]

***Supplementary material***

**Supplementary Figure. 1 Identification of 138 Plasma Proteins Significantly Associated with Primary Knee Osteoarthritis by Circular Heatmap Analysis.**

**Supplementary Figure. 2 Protein - Protein Interaction (PPI) Network Connecting Identified Causal Proteins and Implicated Protein Targets.**

**Supplementary Figure. 3 Findings on the Types of Interactions in Drug - Gene Interaction Analysis.**

**
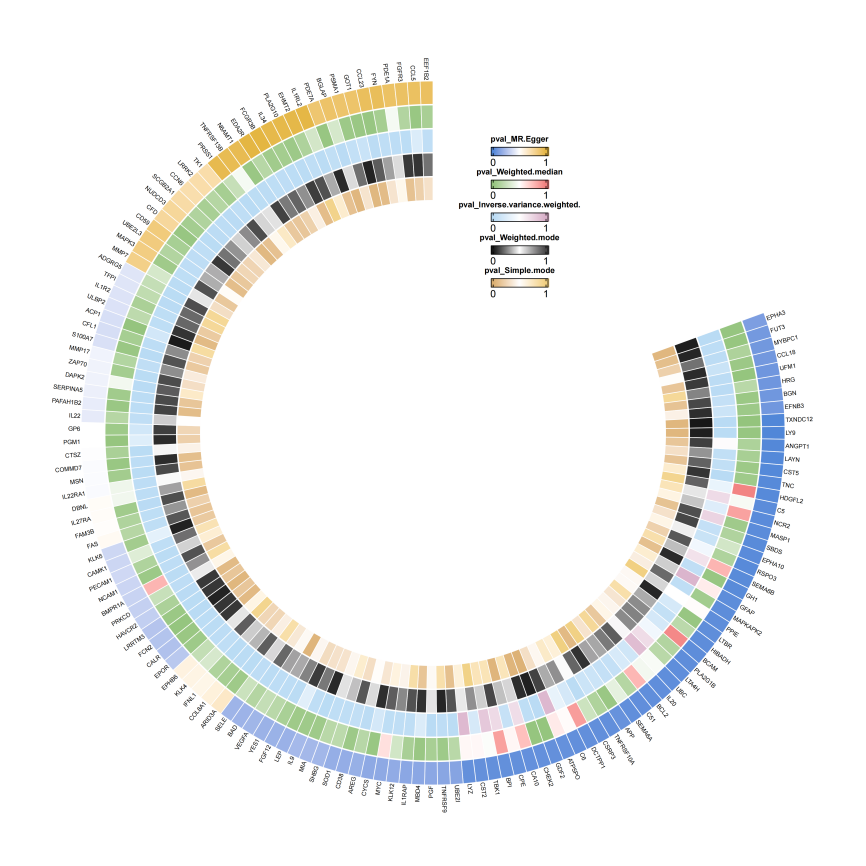
**

**Supplementary Figure. 1 Identification of 138 Plasma Proteins Significantly Associated with Primary Knee Osteoarthritis by Circular Heatmap Analysis.**

**
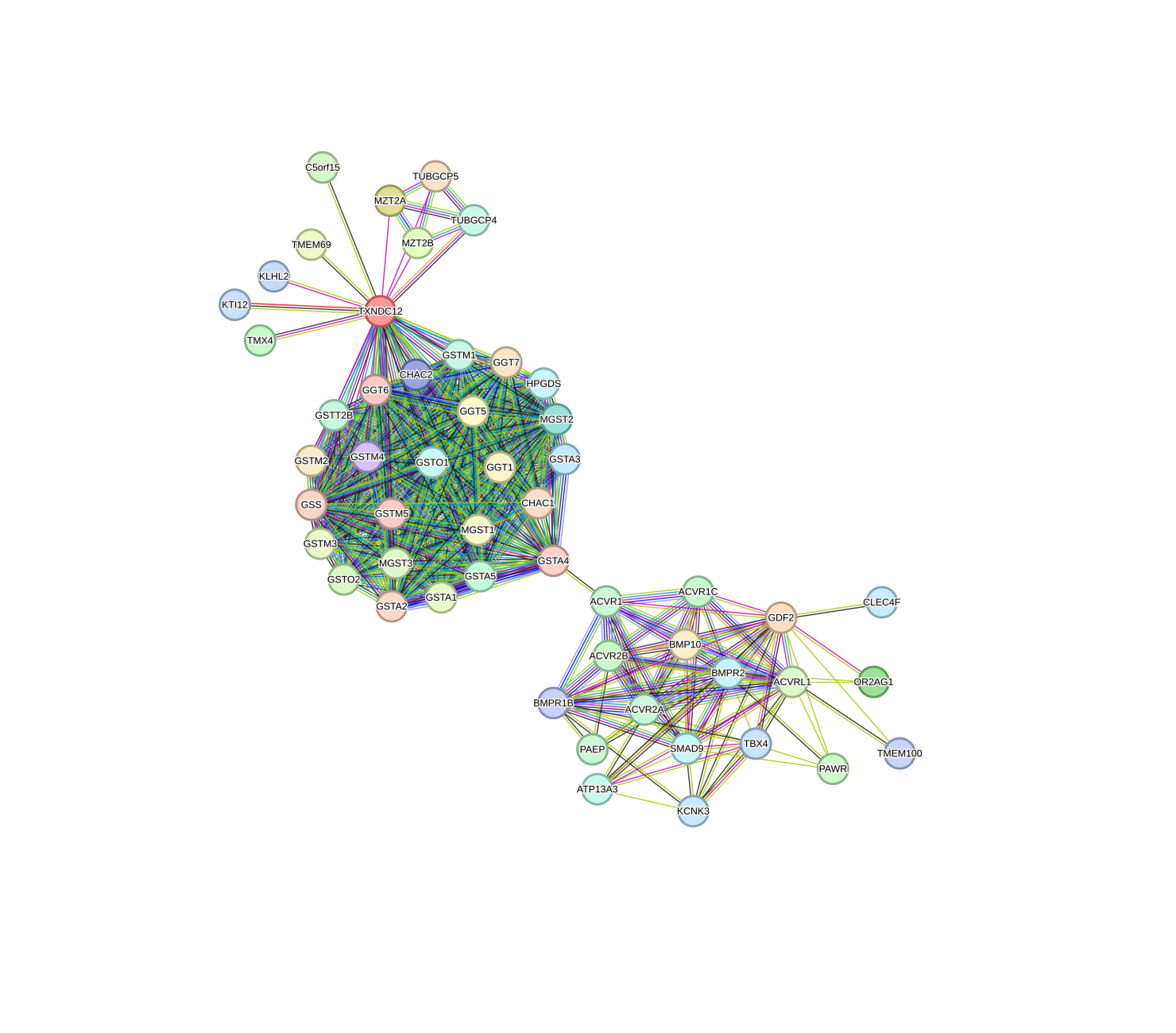
**

**Supplementary Figure. 2 Protein - Protein Interaction (PPI) Network Connecting Identified Causal Proteins and Implicated Protein Targets.**

(a) GSTA3 (b) GSTM4


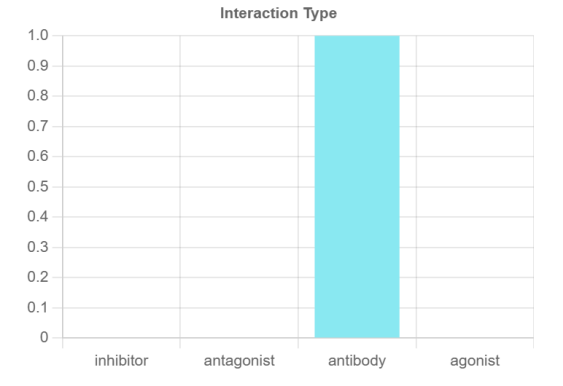

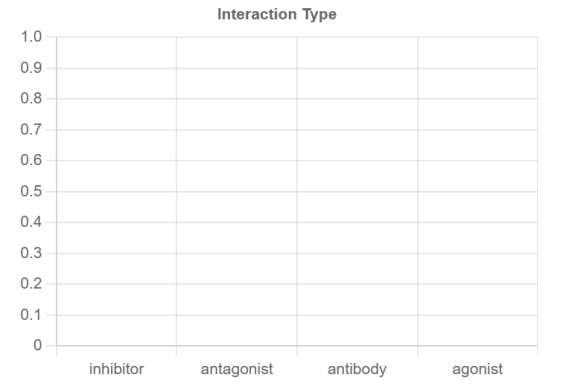


(c) GGT7 (d) GSS


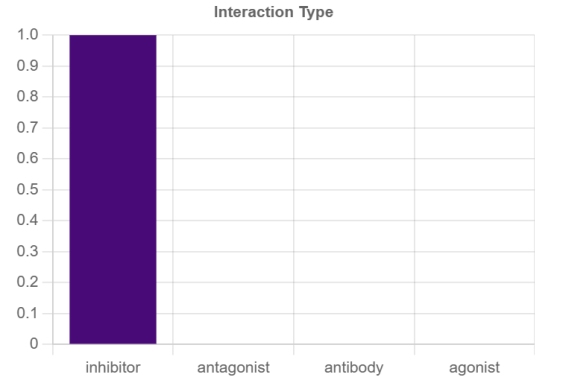

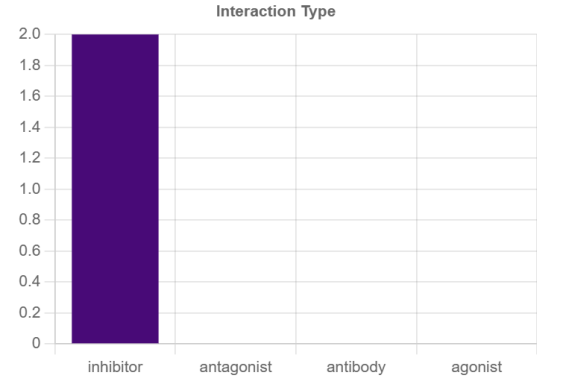


(e) GSTA2 (f) GSTA1


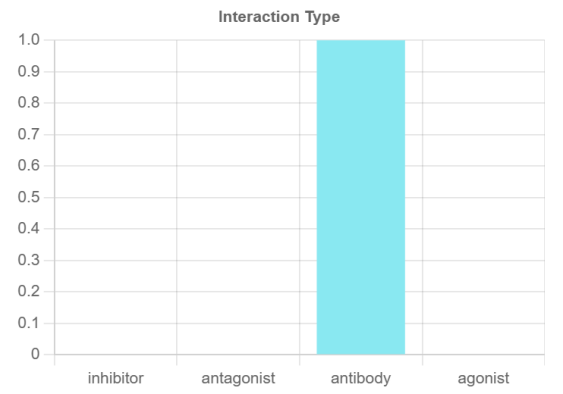

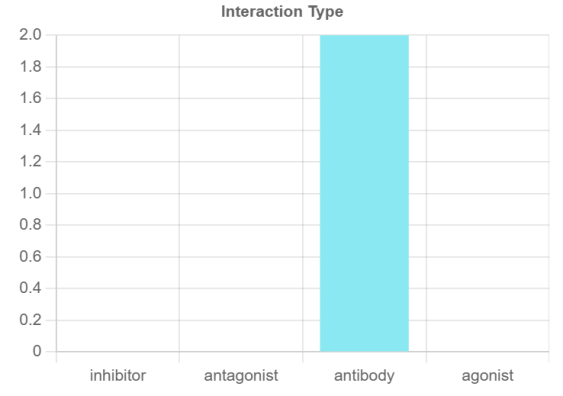


(g) GSTO1 (h) HPGDS


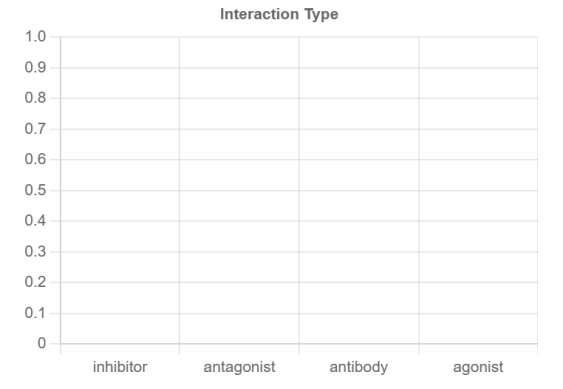

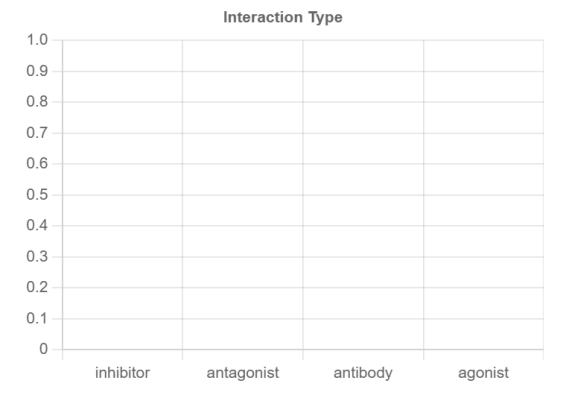


(i) GGT1 (j) GSTM3


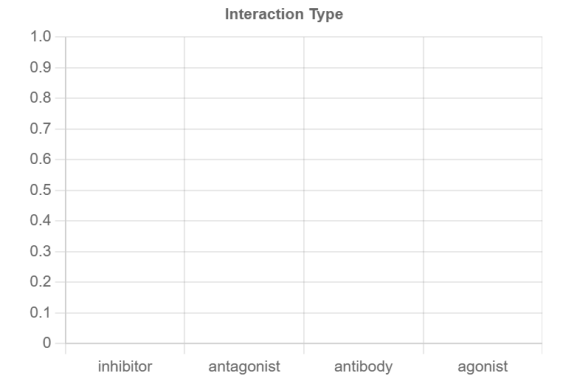

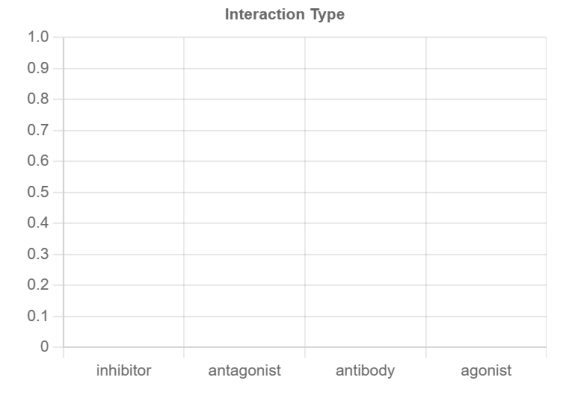


(k) GSTM1 (l) ACVRL1


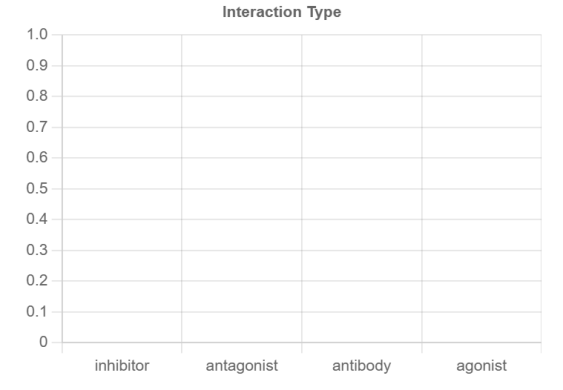

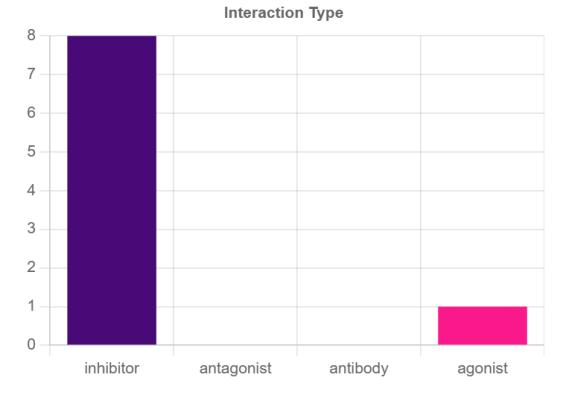


(m) BMPR2 (n)ACVR2A


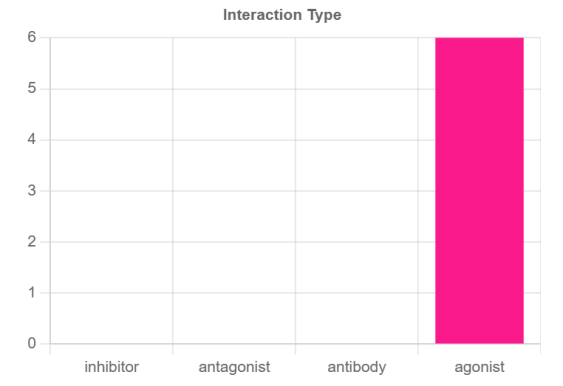

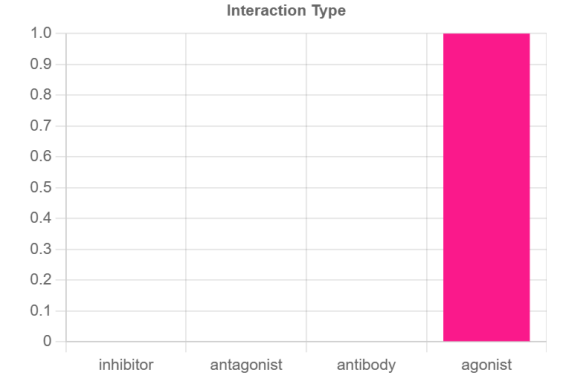


(o) ACVR2B (p) ACVR1


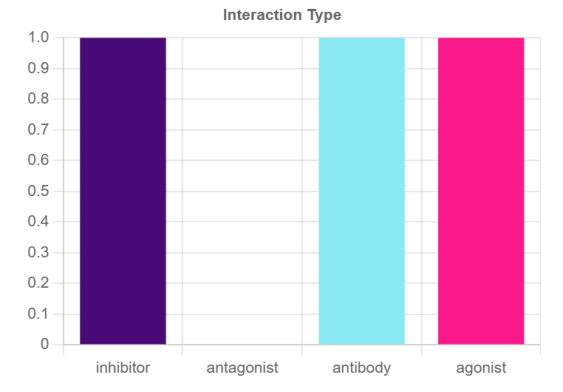

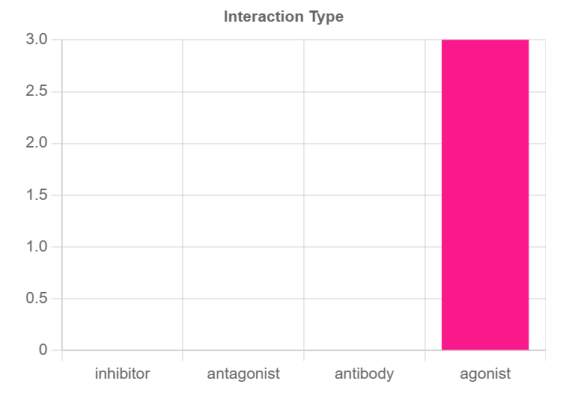


(q) BMP10 (r) BMPR1B


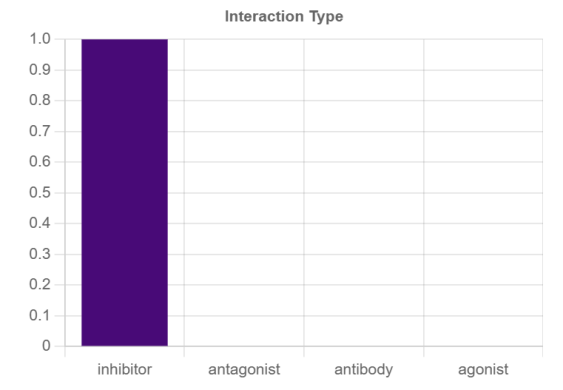

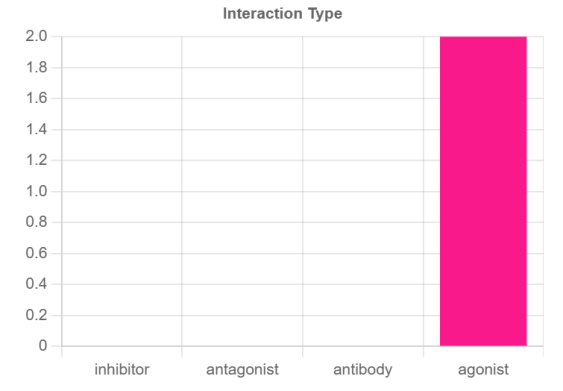


(s) PAEP (t) ACVR1C


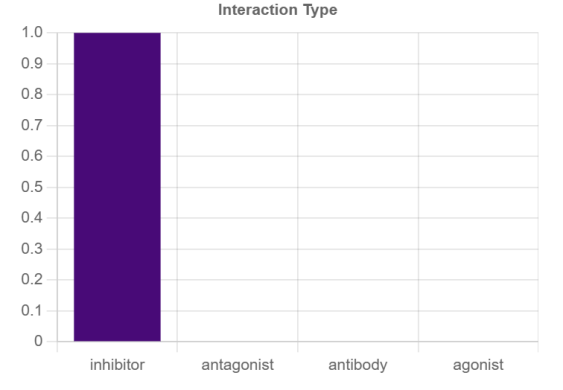

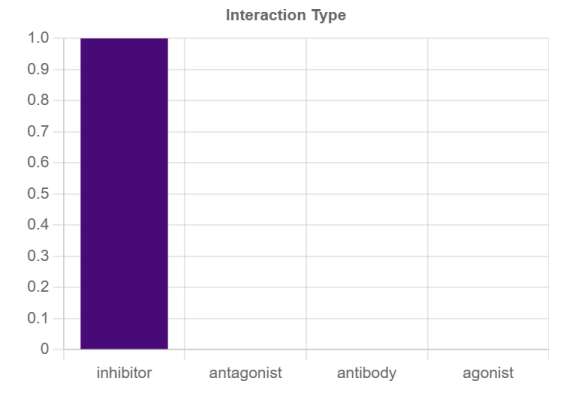


(u) KCNK3


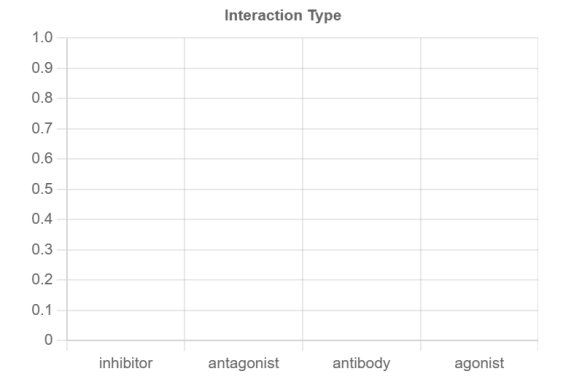


**Supplementary Figure. 3 Findings on the Types of Interactions in Drug - Gene Interaction Analysis. (a)** GSTA3= Glutathione S-transferase A3; **(b)** GSTM4=Glutathione S-transferase Mu 4; **(c)** GGT7=Glutathione hydrolase 7 heavy chain; **(d)** GSS=Glutathione synthetase; **(e)** GSTA2=Glutathione S-transferase A2; **(f)** GSTA1=Glutathione S-transferase A1; **(g)** GSTO1=Glutathione S-transferase omega-1; **(h)** HPGDS=Hematopoietic prostaglandin D synthase; **(i)** GGT1=Glutathione hydrolase 1 heavy chain; **(j)** GSTM3=Glutathione S-transferase Mu 3; **(k)** GSTM1=Glutathione S-transferase Mu 1; **(l)** ACVRL1=Serine/threonine-protein kinase receptor R3; **(m)** BMPR2=Bone morphogenetic protein receptor type-2; **(n)** ACVR2A=Activin receptor type-2A; **(o)** ACVR2B=Activin receptor type-2B; **(p)** ACVR1=Activin receptor type-1; **(q)** BMP10=Bone morphogenetic protein 10; **(r)** BMPR1B=Bone morphogenetic protein receptor type-1B; **(s)** PAEP=Progestagen-Associated Endometrial Protein; **(t)** ACVR1C=Activin receptor type-1C; **(u)** KCNK3=Potassium channel subfamily K member 3.
